# High‐throughput membrane‐anchored proteome screening reveals PIEZO1 as a promising antibody‐drug target for human esophageal squamous cell carcinoma

**DOI:** 10.1002/cam4.4744

**Published:** 2022-05-24

**Authors:** Xun Qin, Zhen Ni, Jianjun Jiang, Xiguang Liu, Xiaoying Dong, Mei Li, Kai Miao, Shuan Rao, Wenqing Zhang, Kaican Cai

**Affiliations:** ^1^ Department of Developmental Biology, School of Basic Medical Sciences Southern Medical University Guangzhou China; ^2^ Department of Thoracic Surgery, Nanfang Hospital Southern Medical University Guangzhou Guangdong China; ^3^ Cancer Center, Faculty of Health Science University of Macau Macau SAR China; ^4^ Division of Cell, Developmental and Integrative Biology, School of Medicine South China University of Technology Guangzhou China

**Keywords:** antibody‐drug conjugate, esophageal squamous cell carcinoma, PIEZO1

## Abstract

**Background:**

Esophageal carcinoma is one of the most fatal cancers worldwide. In China, over 90% of esophageal cancer patients are diagnosed with esophageal squamous cell carcinoma (ESCC). Currently, the survival and prognosis of ESCC patients are not satisfying because of insufficient early screening and lack of efficacious medication. Accumulating studies have suggested that antibody‐drug conjugates (ADC) represent a promising antitumor strategy.

**Method:**

Here, we carried out a specific membrane proteome screening with ESCC patients' samples using a high‐throughput antibody microarray to uncover potential targets for ADC development. Candidates were validated with expression and cytotoxicity evaluation both in vitro and in vivo.

**Results:**

Our data have shown that the Piezo‐Type Mechanosensitive Ion Channel Component 1 (PIEZO1) is particularly overexpressed in human ESCC tumors and can be efficiently internalized when bound with its monoclonal antibody. Furthermore, the PIEZO1 antibody combined with monomethyl auristatin E (MMAE) can specifically kill PIEZO1 high‐expressed ESCC tumor cells by inducing cell cycle arrest and apoptosis. More importantly, the Anti‐PIEZO1‐MMAE can significantly suppress tumor progression in ESCC xenograft tumor models without any obvious side effects.

**Conclusion:**

Taken together, our work demonstrates that PIEZO1 is a promising target to develop ADCs for human ESCC treatment, providing a new strategy for ESCC patients' personalized therapy.

## Graphical abstract

Piezo‐Type Mechanosensitive Ion Channel Component 1 (PIEZO1) is significantly over‐expressed in a large proportion (50%) of ESCC patients. Tumor cell surface located PIEZO1 could be internalized with the binding of its monoclonal antibody. Anti‐PIEZO1‐MMAE can induce cell cycle arrest and apoptosis of ESCC cells in vitro and suppress ESCC tumor cell growth in vivo without any obvious side effect

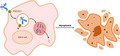

## 1 INTRODUCTION

Esophageal carcinoma is ranked as the 7th most prevalent cancer with roughly 572,000 new cases and the 6th fatal cancer with about 509,000 dead cases annually. The highest incidence of esophageal cancer is recognized in East Asian countries.^1^ By 2020, the prevalence rank of esophageal carcinoma has become the 10th with 604,100 new cases, yet the number of death cases of esophageal cancer ranked the 6th among various cancers with 544,076 deaths, indicating extreme malignancies and inefficient treatment of this very cancer type.^2^ In China, more than 90% of esophageal patients are defined as esophageal squamous cell carcinoma.^3^ Due to insufficient early screening, most ESCC patients showed very malignant clinical and pathological features when diagnosed, making their prognosis and survival relatively poorer than many other cancer patients.^4^ It is known that numerous external factors can contribute to ESCC development and the molecular pathology of ESCC is not fully understood, thus targeted medications rarely benefit ESCC patients.^5^ Although regular treatment options are available including endoscopic resection, chemotherapy, radiotherapy, chemoradiotherapy as well as some targeted medications, the overall outcome is still unsatisfying.^6^ Currently, various tyrosine kinase inhibitors including small molecular antineoplastic drugs and monoclonal antibodies targeting EGFR, HER2, VEGFR, and c‐Met pathways have been developed and tested in human ESCC treatment.^7^ It has been reported that monoclonal antibodies such as cetuximab and nimotuzumab targeting EGFR are useful in advanced ESCC treatment, especially when combined with other therapies.^8^, ^9^ Moreover, clinical trials with immunotherapy targeting PD‐1 by monoclonal antibodies have also shown promising results in treating ESCC.^10^, ^11^ However, the application of these therapies is limited due to drug resistance, pathway crossovers, and tumor cell heterogeneity. More specific targets and therapeutic strategies are in urgent need. Therefore, it is still highly demanded more efficient and specific therapies to improve ESCC patients' prognosis.

Recently, targeting tumor surface antigens with antibody‐drug conjugate has shown great potential in treating various cancers.^12^, ^13^ ADCs targeting special cancers such as brentuximab vedotin for lymphoma,^14^, ^15^ inotuzumab for acute lymphoblastic leukemia,^16^, ^17^ gemtuzumab for acute myeloid leukemia,^18^, ^19^ polatuzumab^20^ and rituximab^21^ for B‐cell lymphoma, and ado‐trastuzumab emtansine for HER2‐positive breast cancer^22^, ^23^ have been approved by FDA for application. An ADC complex consists of a monoclonal antibody which targets a surface protein on tumor cells and is covalently integrated with a cytotoxic agent.^24^ Theoretically, ADCs can recognize the tumor surface antigen and bind to tumor cells specifically, then bring the cytotoxic agent into the tumor cells and subsequently induce cell death.^25^ An ideal ADC antigen should be particularly enriched on the tumor cells’ surface and can be internalized when bound with its monoclonal antibody.^26^ For instance, EGFR has been identified as a potential ADC target for several cancer treatments including ESCC.^27^

In the past, most ADC targets’ development was restricted within well‐characterized membrane‐anchored proteins on tumors, which might miss the novel or unknown targets.^28^ Proteome Epitope Tag Antibody Library array is a powerful platform for high‐throughput and multiplexed protein profiling with a collection of immobilized antibodies targeting different cellular components.^29^ Proteome members located on the cell membrane can be screened directly on the antibody microarray in either a single‐blind (antibody with known target) or double‐blind (antibody with unknown target) manner, which is a great advantage in seek of novel ADC targets.

In this study, we took advantages of the Proteome Epitope Tag Antibody Library (PETAL) which included more than 60,000 monoclonal antibodies to carry out overexpressed membrane protein screening between human ESCC tumor and paracancerous normal tissues. Candidates were screened by performing expression and internalization validation tests. We, then, further examined the potential of these antigens as the candidate for ESCC ADC drugs' development through cytotoxicity evaluation both in and ex vivo.

## 2 MATERIALS AND METHODS

### 2.1 Patient sample preparation

PETAL microarray chips were purchased from Abmart. Human ESCC and paracancerous tissues were freshly excised from the same patient who received surgery. Tissues were rinsed in ice‐cold PBS to remove blood cells after being cut into pieces smaller than 0.5 mm. Tissue sections were then homogenized in PBS 2‐mM EDTA and passed through a 100‐ μM cell strainer. Cell samples were collected via centrifugation at 500*g* for 10 min. The cell membrane was permeabilized with 0.1% TritonX‐100 in PBS 2 mM EDTA for 5 min at 4°C and cytoplasm protein was squeezed out by centrifugation at 5000*g* for 10 min. The cell membrane was then crushed with 0.5% TritonX‐100 in PBS 2 mM EDTA for 30 min at 4°C and membrane protein samples were collected through centrifugation at 12000*g* for 10 min. Protease inhibitor cocktails were added to all the buffers before use.

### 2.2 Cell culture

Human ESCC cell lines including TE‐1, Kyse150, and Eca109 were purchased from the Chinese Academy of Science. Cells were cultured at 37°C with 5% CO_2_ in Dulbecco's modified Eagle's medium (Gibco, C11995500BT) with 10% FBS according to the providers' recommendation.

### 2.3 Protein immunoblotting

Membrane protein samples were isolated and boiled in protein loading buffer for 10 min. For PIEZO1, 8% SDS PAGE was performed and protein was transferred to nitrocellulose membrane at 250 mA for 6 h. Anti‐PIEZO1 (1:500, Proteintech, 15,939‐1‐AP) was applied in TBST 5% milk overnight at 4°C and anti‐Rabbit IgG‐HRP (1:5000, Proteintech, SA00001‐2) was incubated for 2.5 h at room temperature. ACTB antibody (1:5000, Proteintech, 66,009‐1‐Ig) was used as internal control and anti‐mouse IgG‐HRP (1:5000, Proteintech, SA00001‐1) was applied as recommended. Other protein immunoblotting was conducted accordingly. Bands were detected and visualized with ECL reagent (Thermo Fisher, 32,109). In consideration of the huge gap of stripe strength between PIEZO1 and the internal control, the membrane was cut and developed, respectively.

### 2.4 PETAL array screening and analysis

Membrane proteomic samples were isolated from human ESCC tumors and paracancerous tissues and controlled by immunoblotting of GAPDH (Abcam, ab9485) and ATPSB (Abmart, M40013M). Qualified protein samples were labeled with biotin using EZ‐Link NHS‐LC‐Biotin (Thermo Fisher, 21,366) and applied to the PETAL array. A secondary fluorescent antibody carrying Cy3 was hybridized with the array subsequently. The fluorescent intensity of each antibody spot was screened and normalized with internal control. A fluorescent intensity fold change of 1.5 or higher was considered significant. Tumor‐specific highly expressed antigens on the membrane selected from different patients were then overlapped to identify general overexpressed membrane proteins for further validation. The R program was used to draw a Venn diagram.

### 2.5 Internalization evaluation of antibodies

Cells were seeded per plate and incubated with primary antibody (10 μg/ml) for 30 min at 4°C, then cultured under 37°C for 4 h. Antibody attached cells were rinsed three times with PBS and fixed with 4% PFA at room temperature for 10 min. A fluorescent secondary antibody was then incubated for 1.5 h. DAPI was used for nuclei staining. Images were taken with Zeiss 800 confocal system. Cells were also collected and analyzed with flow cytometry detection. Surface mean fluorescence intensity (MFI) was calculated and compared.

### 2.6 Immunofluorescence and immunohistochemistry

For immunofluorescence, fixed cells were incubated with primary antibody (1:200) at 4°C overnight, and then, fluorescent secondary antibody (1:500) was incubated for 1.5 h at room temperature. Cell nuclei were stained with DAPI. Images were acquired with Zeiss 800 confocal system. Immunohistochemistry was carried out on tissue sections of patients' samples and two tissue array chips. Sections and tissue array chips were heated at 65°C for 4 h, then dewaxed in xylene and dehydrated in ethanol. Antigen retrieval was achieved by microwaving slides in citrate buffer. The primary antibody was incubated for 2 h at room temperature and the secondary antibody labeled with HRP was incubated for 1 h, after which DAB staining was conducted. IHC signal was read under light microscopy and scored based on staining intensity as “−” for negative, “+” for low, “++” for moderate, and “+++” for high. Ki67 staining was evaluated with tumor tissues harvested from xenograft models. Positive cells were counted from at least five fields and compared.

### 2.7 Isolation of total RNA and RT‐qPCR assay

Total RNA was isolated using the phenol–chloroform method as previously described.^30^ Reverse transcription was done according to standard routine (Toyobo, TRT‐101). PCR reaction was conducted with Roche LightCycler 96. PCR primers were as follows: PIEZO1 (sense: 5′‐GGA CTC TCG CTG GTC TAC CT‐3′; anti‐sense: 5′‐ GGG CAC AAT ATG CAG GCA GA‐3′), ACTB (sense: 5’‐CAC CAT TGG CAA TGA GCG GTT C‐3′; anti‐sense: 5′‐GGT CTT TGC GGA TGT CCA CGT‐3′).

### 2.8 In vitro antitumor effect evaluation

ESCC cell lines were seeded per well and incubated with 0–100‐nM Anti‐PIEZO1‐MMAE for 48 h. Cell growth was evaluated with CCK‐8 assay (Beyotime, C0038). One hundred microliters of CCK‐8 reaction buffer was added into each well and incubated at 37°C for 30 min. OD450 was acquired by Synergy HTX multi‐mode reader. The cell viability ratio was calculated and normalized to untreated control. IgG‐MMAE and Anti‐PIEZO1 were used as isotype control and vehicle control, respectively.

### 2.9 Cytometry analysis of cell apoptosis and cycle

Cell samples were harvested using trypsin and washed with PBS for three times. Apoptosis and cell cycle distribution were detected through flow cytometry analysis of Annexin V staining and PI staining (Solarbio, CA1020) according to the standard routine. Living (Annexin V^−^/PI^−^), early apoptotic (Annexin V^+^/PI^−^), late apoptotic (Annexin V^+^/PI^+^), and necrotic (Annexin V^−^/PI^+^) cells were distinguished. Data were analyzed by FlowJo.

### 2.10 ESCC tumor models in NOD/SCID mice

To establish tumor models in NOD/SCID mice, 1 × 10^7^ TE‐1 cells were suspended in Matrigel (BD, 354234) and injected subcutaneously in the right armpit. The treatment of Anti‐PIEZO1‐MMAE or control reagents started when the tumor volume reached 300 mm^3^. Treatments for each group were given every 3 days in total four times. Five mice were involved per group. Tumor volume and body weight were recorded and compared among groups.

### 2.11 Statistical analysis

Each experiment was repeated for more than three times. Independent Student's *t*‐test was used to examine the significance of differences between the two groups. The chi‐squared test was used to evaluate the correlation between PIEZO1 expression level and chosen clinicopathologic parameters. Statistical analysis was performed by SPSS 19.0. The mean and standard deviation were shown. A two‐tailed p‐value less than 0.05 was considered significant. *p value <0.05, **p‐value <0.01, ***p‐value <0.001, ****p‐value <10^−4^.

## 3 RESULTS

### 3.1 Identification of PIEZO1 as a potential ADC target for human ESCC

**FIGURE 1:**
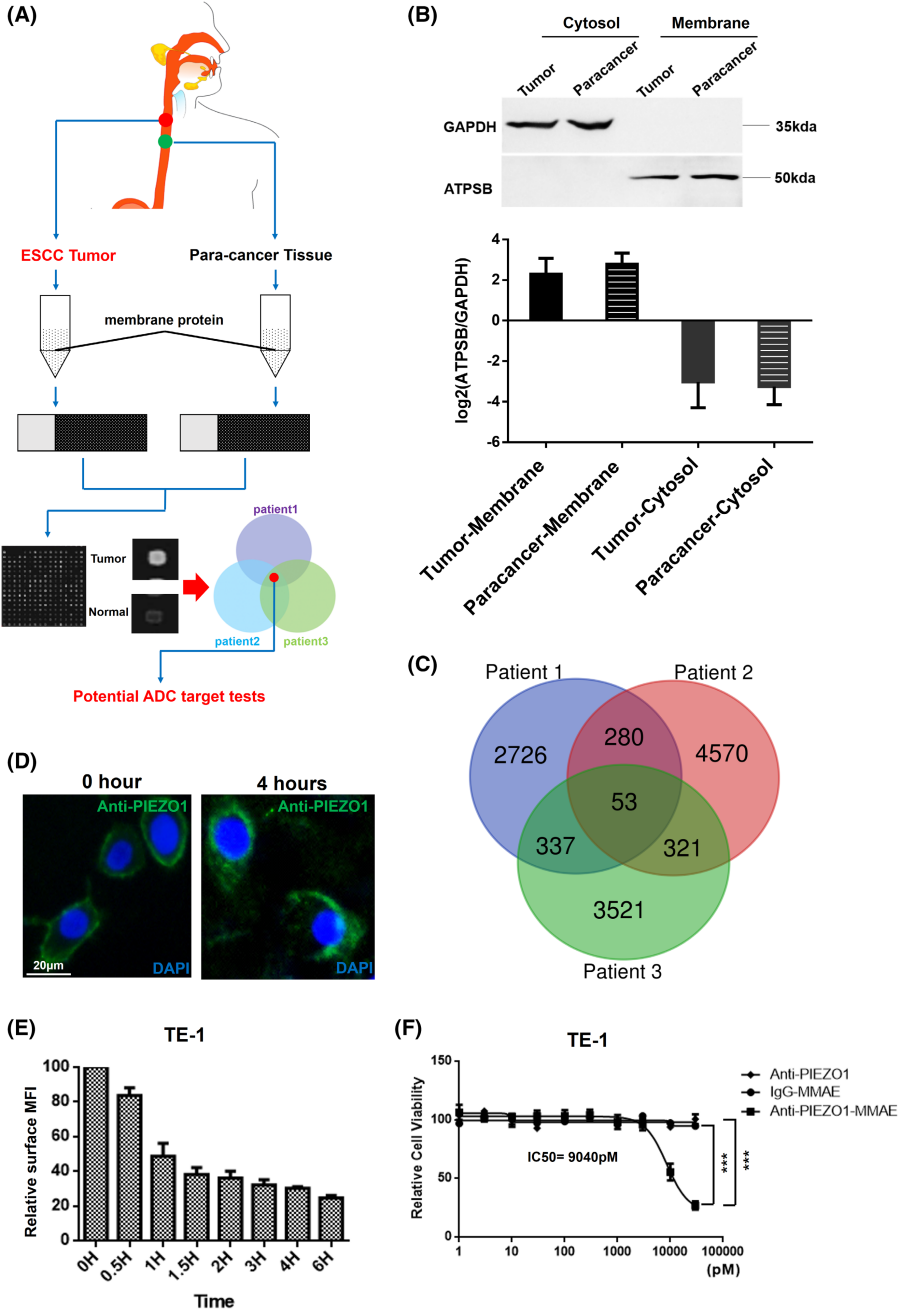
PIEZO1 is identified as a potential ADC target by high‐throughput membrane proteome screening. (A) The schematic diagram for the whole study design; (B) The quality control of membrane‐anchored proteins' enrichment derived from ESCC patients' samples, GAPDH was used for defining cytosol proteins and ATBSB was for membrane components; (C) Overlapping of overexpressed membrane protein candidates between and among patients; (D) Internalization assay by immunofluorescence using PIEZO1 antibody in TE‐1 cells (scale bar: 20 μm); (E) Internalization efficiency evaluation of PIEZO1 antibody into TE‐1 cells with flow cytometry quantification of PIEZO1 immunofluorescence; (F) Cytotoxicity test of Anti‐PIEZO1‐MMAE in TE‐1 cells, Anti‐PIEZO1 antibody, and IgG‐MMAE were used as vehicle and isotype control, respectively

To search for potential ADC targets for ESCC, we firstly carried out overexpressed membrane protein screening in three individual ESCC patients' samples using antibody microarrays according to standard protocol (Figure 1A). Membrane protein sample isolation was controlled by relatively enriched protein marker ATPSB and GAPDH was used for cytosol component control (Figure 1B). In our study, both tumor tissue and paracancerous tissues were utilized. Fluorescent intensity fold changes of 1.5 or higher were considered significant. With this threshold, the tumor‐specific overexpressed membrane proteins were selected and compared among all patients (Figure 1C). Not surprisingly, our results indicated a huge variation of membrane protein profiles among different ESCC patients on account of the extraordinary esophageal tumor heterogeneity (Figure S1A). Subsequently, the most commonly overexpressed and a subset of two patients' highly expressed membrane proteins, including in total of 180 candidates, were selected for further ADC targets’ validation. Cell internalization assays of candidate antibodies were conducted within human ESCC cell lines TE‐1. Among the 180 selected candidates, PIEZO1 was identified as the most promising candidate as it showed strong internalization capacity with the binding of its monoclonal antibody (Abmart, M25233) at physiological condition (Figure 1D). Our data showed that nearly 80% of cell surface PIEZO1 could be internalized within 6 h compared to isotype control (Figure 1E), which was a great advantage for PIEZO1 as an ADC target. PIEZO1 was only overexpressed in patient 1 and patient 2 but not in patient 3 (Figure S1B), indicating heterogeneous expression of PIEZO1 among patients. Next, by combining the specific antibody and a classical antineoplastic agent, monomethyl auristatin E (MMAE), we investigated whether these ADCs could kill ESCC cells efficiently. ADCs were obtained via commercial synthesis and verified. TE‐1 cells were seeded and incubated with ADCs for 48 h and analyzed for cell viability. Indeed, Anti‐PIEZO1‐MMAE showed tremendous toxicity to TE‐1 even at very low concentrations (IC50: 9040pM), whereas Anti‐PIEZO1 alone or IgG‐MMAE had no tumor suppression effect at the same concentrations (Figure 1F). Furthermore, we tested a few other ADC candidates which were also highly expressed on tumor cell membrane; our data indicated that Anti‐PIEZO1‐MMAE had a much more significant tumor‐suppressing activity than any other ADCs (Figure S1C).

### 3.2 PIEZO1 is overexpressed in ESCC patients' tumors

**FIGURE 2:**
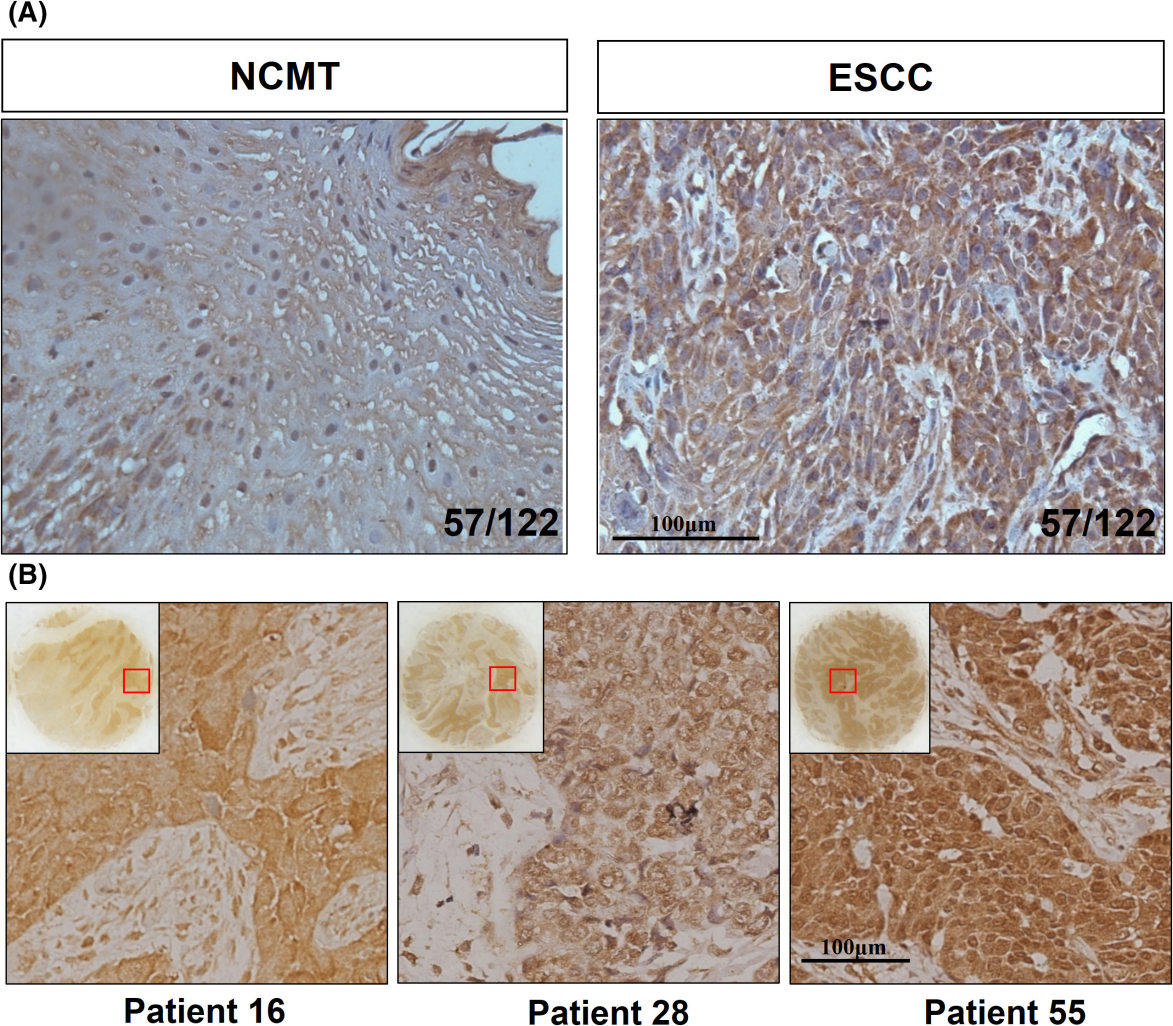
PIEZO1 is overexpressed in human ESCC tumor cells. (A) IHC detection of PIEZO1 expression in tumor and normal tissues with in‐house collected ESCC patients' samples (magnification: 200×, scale bar: 100 μm); (B) Representative expression patterns of PIEZO1 in human ESCC samples (magnification: 200×, scale bar: 100 μm)

To validate our previous finding that PIEZO1 could be a promising ADC candidate for human ESCC cells, the evaluation of PIEZO1 expression in a larger number of human ESCC samples is required. To date, PIEZO1 is rarely studied in human ESCC cells. Only one study demonstrated that PIEZO1 was overexpressed in ESCC tumors compared to normal tissue, in which only 49 tumors and 25 normal tissues were involved and evaluated in unpaired test, thus could not determine the difference of PIEZO1 expression between tumor and normal tissues in the same patient. Therefore, we explored PIEZO1’s expression pattern and its relevance to ESCC patients' prognosis in a larger patient population. We performed in‐house PIEZO1 IHC analysis of 56 pairs of ESCC patients' tumor and paracancerous tissues as well as two tissue arrays including 162 ESCC patients' tumors and 66 corresponding normal tissues. Samples with staining intensity of “+++” were considered PIEZO1‐high, others PIEZO1‐low. Our results showed that PIEZO1 was significantly overexpressed in ESCC tumors (Table 1). Further statistical analysis showed that 80% (97/122) of ESCC patients' tumor tissues overexpressed PIEZO1, about 50% (57/122) of ESCC patients had a negative or weak expression of PIEZO1 in paracancerous normal tissue but the high expression in tumor tissue, indicating a considerable proportion of patients suitable for PIEZO1 antibody‐conjugate drugs (Figure 2A). Of note, we observed that PIEZO1 could be detected on the cell membrane, nuclear membrane, and in the cytoplasm in different patients (Figure 2B). Further analysis of the correlation between PIEZO1 expression level and clinicopathologic parameters was conducted on 162 ESCC patients with clinical information. Albeit the expression level and pattern of PIEZO1 showed significant heterogeneity among different patients, our result showed that the expression of PIEZO1 had no correlation with ESCC tumor stage and progression, suggesting that targeting PIEZO1 with ADC would not bring any additional tumor facilitating effect (Table 2). In addition, transcriptome profiles of 93 ESCC and 13 control samples were acquired from the TCGA database and re‐analyzed. The result also showed that PIEZO1 was significantly overexpressed in human ESCC tumors than adjacent normal tissues at the transcriptional level (Figure S2A). Similarly, no association was found between the PIEZO1’s expression level and tumor stage, tumor progression, and overall survival of ESCC patients (Figure S2B–D), in accordance with our previous conclusion with tissue arrays.

**TABLE 1:** PIEZO1 expression in ESCC tumor and paracancerous tissue

ESCC, *n* = 122			
	Tumor	Normal	*p*‐value
−	9	33	<0.0001
+	11	74	
++	33	13	
+++	69	2	

**TABLE 2:** Relation of PIEZO1 with clinical characters of ESCC patients

ESCC, *n* = 162				
		PIEZO1‐high	PIEZO1‐low	*p*‐value
Stage(T)	1	4	1	0.237
	2	12	15	
	3	59	63	
	4	2	0	
Stage(N)	0	44	36	0.170
	1	21	32	
	2	13	11	
	3	4	1	
Stage(M)	0	82	80	1
	1	0	0	
Grade	I	7	6	0.610
	I–II	19	18	
	II	28	34	
	II–III	7	8	
	III	20	12	
Sex	M	49	56	0.784
	F	33	24	

### 3.3 The lethal effect of Anti‐PIEZO1‐MMAE is dependent on PIEZO1 expression

**FIGURE 3:**
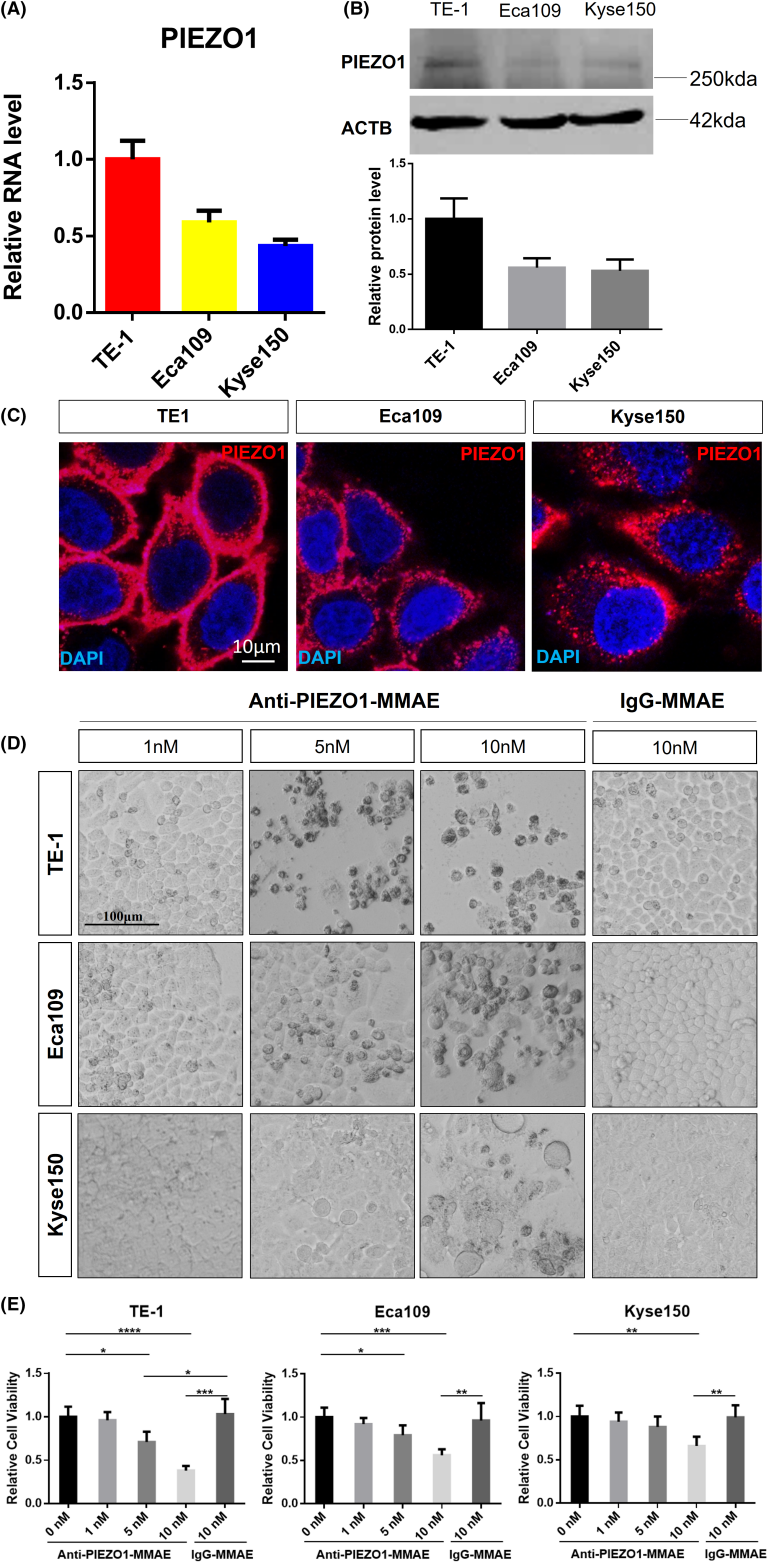
The cytotoxicity of Anti‐PIEZO1‐MMAE is the dosage and cell surface PIEZO1 expression dependent. (A, B) Transcription and protein level of PIEZO1 in human ESCC cell lines TE‐1, Eca109, and Kyse150. ACTB was used as an internal control. (C) In situ immunofluorescence staining assay of PIEZO1 in TE‐1, Eca109, and Kyse150, scale bar: 100 μm; (D) Microscopic assay of cell morphology of TE‐1, Eca109, and Kyse150 treated with different dosages of Anti‐PIEZO1‐MMAE (1, 5, and 10 nM) for 48 h; IgG‐MMAE was used as isotype control, scale bar: 100 μm; (E) Cell viability assay via CCK‐8 test of TE‐1, Eca109, and Kyse150 treated with different dosage Anti‐PIEZO1‐MMAE (1, 5, and 10 nM) for 48 h; data were normalized to empty control

To evaluate the efficacy and specificity of Anti‐PIEZO1‐MMAE, we employed two other human ESCC cell lines Kyse150 and Eca109 in addition to TE‐1. We detected PIEZO1’s expression at both RNA and protein levels. Not surprisingly, the expression of PIEZO1 was quite different among different ESCC cell lines, with the strongest expression in TE‐1 cells and lower expression in Eca109 and Kyse150 cells (Figure 3A,B). Moreover, the immunofluorescence assay indicated that not only the expression of PIEZO1, but also the cellular distribution was slightly distinct among different cell lines. With no big difference in expression level, PIEZO1 was more enriched in the cytoplasm and less enriched on the cell membrane than Eca109 cells (Figure 3C). We, then, evaluated the cytotoxic effect of Anti‐PIEZO1‐MMAE on TE‐1, Eca109, and Kyse150 cells using IgG‐MMAE as the control. Cells were incubated with Anti‐PIEZO1‐MMAE and control reagent for 48 h. Microscopic observation showed that TE‐1 was quite sensitive to Anti‐PIEZO1‐MMAE treatment, as most cells exhibited apoptotic morphology when incubated with 10‐nM Anti‐PIEZO1‐MMAE. In contrast, the other two cell lines, especially Kyse150, which had lower expression and less cell surface enrichment of PIEZO1, were more resistant to Anti‐PIEZO1‐MMAE treatment than TE‐1, suggesting ESCC cells with higher cell surface PIEZO1 expression were more sensitive to Anti‐PIEZO1‐MMAE treatment (Figure 3D,E). Importantly, IgG‐MMAE had no toxic effect on any ESCC cells at experimental concentrations, further supporting the notion that the lethal effect of Anti‐PIEZO1‐MMAE on ESCC cells was PIEZO1 expression dependent.

### 3.4 Anti‐PIEZO1‐MMAE induces apoptosis and cell cycle arrest in ESCC cells

**FIGURE 4:**
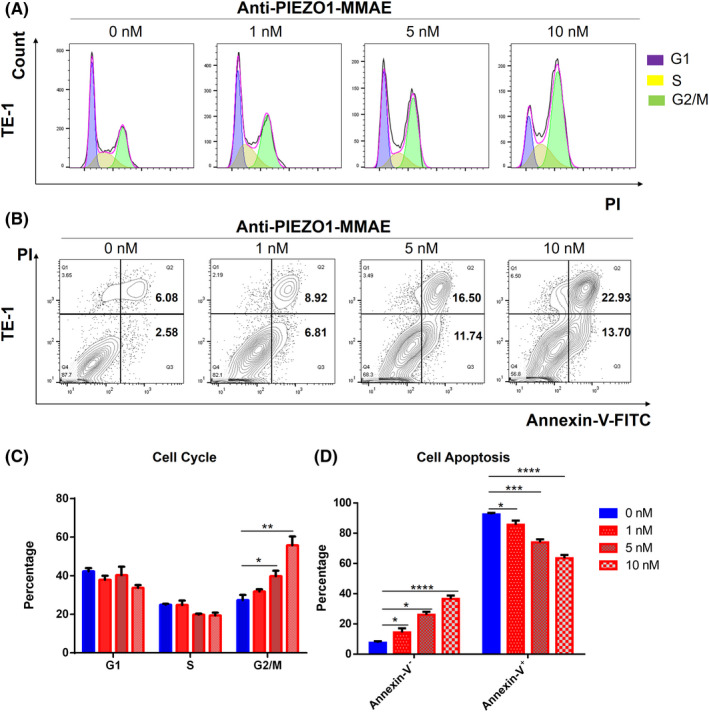
Anti‐PIEZO1‐MMAE induces cell cycle arrest and apoptosis in TE‐1 cells. (A) Cell cycle analysis of TE‐1 cells treated with Anti‐PIEZO1‐MMAE (1, 5, and 10 nM) for 36 h; (B) Apoptosis analysis of TE‐1 cells treated with different dosages of Anti‐PIEZO1‐MMAE by flow cytometry. (C, D) Quantification of (A, B)

MMAE is reported to be a synthetic derivative of dolastatin 10 and serves as a potent mitotic inhibitor of tubulin polymerization. The introduction of MMAE into proliferative cells can cause severe mitotic defects, leading to cell cycle arrest and apoptosis.^31^ We, then, validated how Anti‐PIEZO1‐MMAE killed ESCC cells. TE‐1 cells were incubated with different concentrations of Anti‐PIEZO1‐MMAE for 36 h and applied to cell cycle and apoptosis assays. The flow cytometry analysis results suggested a clear dosage‐dependent effect of Anti‐PIEZO1‐MMAE to induce G2/M phase arrest in TE‐1 cells (Figure 4A,C). Consistently, higher concentrations of Anti‐PIEZO1‐MMAE also led to more apoptotic and dead cell proportions in TE‐1 cells (Figure 4B,D). Besides, a high concentration (10 nM) of Anti‐PIEZO1‐MMAE could also induce cell cycle arrest and apoptosis in Eca109 and Kyse150 cells (Figure S3). These data collectively indicated that Anti‐PIEZO1‐MMAE could kill ESCC cells via inducing cell cycle arrest and apoptosis.

### 3.5 Anti‐PIEZO1‐MMAE suppresses the growth of ESCC tumors in vivo

**FIGURE 5:**
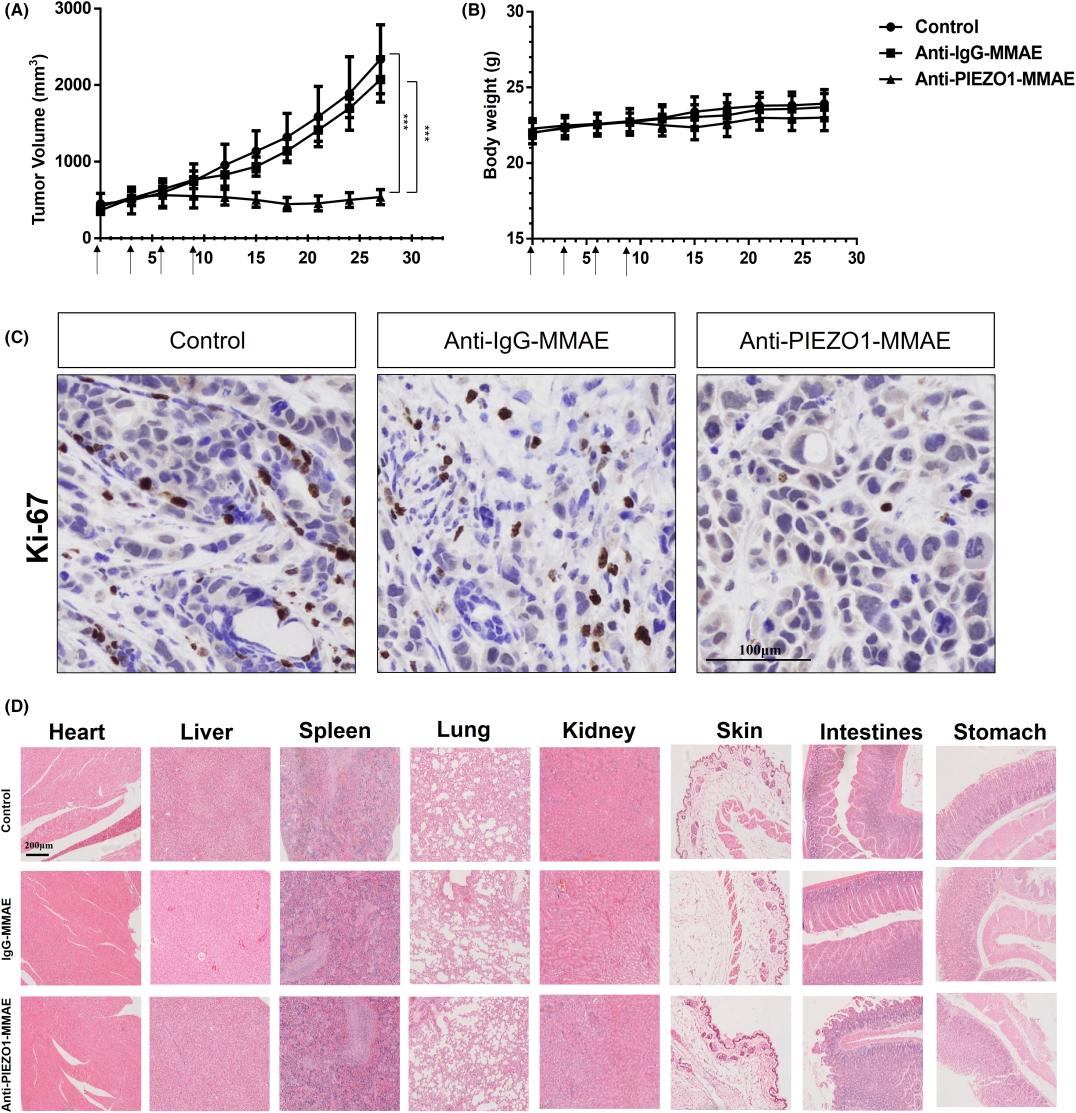
Anti‐PIEZO1‐MMAE shows a strong antitumor effect in vivo without obvious side effects. (A) Tumor growth curve of TE‐1 tumor xenograft. Drug treatment started when the tumor volume reached 300 mm^3^. Mice were treated with Anti‐PIEZO1‐MMAE (5 mg/kg), IgG‐MMAE (5 mg/kg), and PBS, respectively; each mouse was treated every 3 days for in total of four times. *n* = 5 per group; (B) Body weight data of TE‐1 tumor xenograft mice with different treatments; (C) Proliferation evaluation of xenograft tumor cells with Ki‐67 staining (magnification: 200×, scale bar: 100 μm); (D) Histopathological examination of various organs including the heart, liver, spleen, lung, kidney, skin, intestine, and stomach (magnification: 100×, scale bar: 200 μm)

**FIGURE 6:**
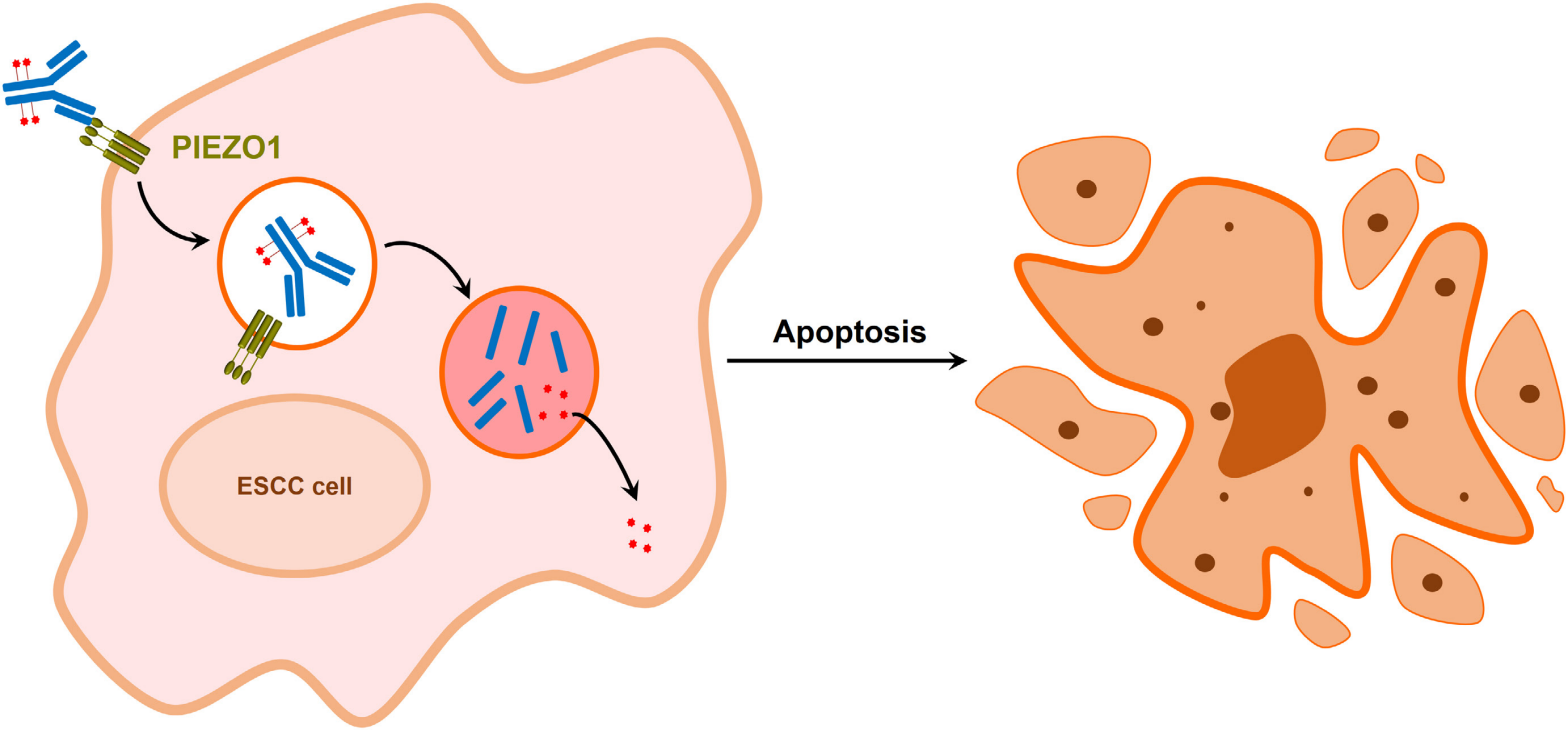
Schematic diagram of how antibody‐drug conjugate targeting PIEZO1 works

To investigate the antitumor effect of Anti‐PIEZO1‐MMAE in vivo, we selected TE‐1 cells to establish a xenograft model in NOD/SCID mice. 1 x 10^7^ TE‐1 cells were suspended in matrigel and transplanted in the right armpit of each mouse. When the tumor volume reached 300 mm^3^, the tumor‐bearing mice were divided into three groups with at least five mice per group and treated with PBS, 5‐mg/kg IgG‐MMAE, and 5‐mg/kg Anti‐PIEZO1‐MMAE, respectively. Drug treatment was given through tail vein injection every 3 days for four times in total. Tumor volume and body weight were monitored every 3 days. Tumor tissues and other organs were collected for histopathological examination when the mice were sacrificed. In line with the in vitro experiments, 5‐mg/kg Anti‐PIEZO1‐MMAE treatment could dramatically suppress tumor development as compared to vehicle and isotype control (Figure 5A) without any significant effect on body weight (Figure 5B). We, then, performed Ki67 staining in the tumor sections from all groups. Not surprisingly, cell proliferation evaluation by Ki67 staining revealed that Anti‐PIEZO1‐MMAE could efficiently inhibit ESCC cells’ propagation in vivo (Figure 5C). Histological examination revealed no obvious side effects with 5‐mg/kg Anti‐PIEZO1‐MMAE treatment in the heart, liver, spleen, lung, kidney, skin, intestine, and stomach (Figure 5D), suggesting that Anti‐PIEZO1‐MMAE is a safe and specific drug candidate against ESCC for future investigation (Figure 6).

## 4 DISCUSSION

ESCC is one of the most common and lethal cancers worldwide, particularly in East Asian countries. So far, the genetic and molecular mechanisms of how ESCC tumors initiate and develop are still not well‐characterized, making it more difficult to treat ESCC patients. Moreover, in consequence of the lack of efficient early screening procedures, most ESCC patients were diagnosed as late stage with local or advanced metastasis.^32^ Currently, surgical resection combined with radiotherapy or chemotherapy is still the main clinical therapeutic option for ESCC patients. The survival and prognosis for ESCC patients are relatively poor as compared to many other cancer patients.^33^ Although researchers have been devoted to exploring the potential of using targeted therapy and immunotherapy for ESCC treatment, their limitations such as drug resistance, mild survival advantage, or severe side effects are already recognized in different clinical trials. In recent years, the antibody‐drug conjugate has emerged as a promising antitumor strategy for various cancers.^34^ For instance, trastuzumab, a conjugate of HER‐2 monoclonal antibody with topoisomerase I inhibitor, has been applied as a first‐line treatment for HER‐2 positive cancer patients^35^, ^36^; however, according to reports, the response rate of trastuzumab ranges was only from 30% to 60% in gastroesophageal cancer.^37^, ^38^ Other available ADCs have been developed in research with advantages and possible limitations discussed.^39^, ^40^ Potential ADC targets’ landscape exploration was tried out across a broad range of tumor types via transcriptome sequence analysis and IHC screening.^41^

The ideal antibody‐drug conjugate is highly specific and hypertoxic as it combines one monoclonal antibody and a small molecular chemical. However, this requires proper ADC targets, which should be significantly overexpressed on the tumor cell surface for antigen recognition and capable of being internalized to guide the drugs into the tumor cells. Albeit with existing ADC targets across different cancer types reported and their priorities and usage discussed,^42^ there is still a huge demand for new ADC targets for human cancer therapy.

Our study aims to discover novel potential ADC targets for human ESCC. Briefly, we extracted and labeled membrane protein components from both human ESCC tumor and paracancerous normal tissues and applied them to antibody microarrays. Tumor overexpressed candidates were then selected and tested with internalization and cytotoxicity evaluation assays in ESCC cell lines. We further identified that the monoclonal antibody targeting PIEZO1 could be efficiently internalized and, therefore, could possibly serve as a potential ADC candidate. As this result was generated from only three ESCC patients' samples, we, then, investigated the PIEZO1 expression using both in‐house ESCC patients' samples and TCGA datasets, by which we found that PIEZO1 was significantly overexpressed in about 50% of ESCC patients. However, correlation analysis shows that PIEZO1 has no effect on ESCC progression and the patients' survival.

Current studies have reported that PIEZO1 is a force‐sensitive cation channel that is critical in maintaining cell homeostasis and normal functions.^43^ Loss of PIEZO1 could result in severe developmental and physiological defects.^44^, ^45^ Researchers have also defined PIEZO1 as an oncogene in multiple human cancers like gastric cancer and glioma.^46^, ^47^ But the role of PIEZO1 in human ESCC has rarely been discovered. A recent study demonstrated that PIEZO1 can bind to TP53 and regulate the cell cycle progression and apoptosis, eventually affecting the tumor growth of ESCC cells.^48^ However, this research lacks solid rescue experiments by interfering with the TP53 function, leaving the conclusion debatable. Therefore, the exact function and mechanism of PIEZO1 in human ESCC remain unclear.

Regardless of the cation channel function, our research has identified PIEZO1 as an endocytosis medium that could be internalized when bound with its antibody. Multiple existing ADC studies have shown that MMAE‐linked ADCs function through cell proliferation depression due to cycle arrest.^49^, ^50^ We further confirmed that Anti‐PIEZO1‐MMAE could very efficiently kill tumor cells via inducing cell cycle arrest and apoptosis, which was highly dependent on the dosage of Anti‐PIEZO1‐MMAE and PIEZO1 expression levels. This result somehow proved that the cytotoxicity of Anti‐PIEZO1‐MMAE was originated from the mitotic inhibitor MMAE, and the specificity was mediated by the PIEZO1 as the isotype control or vehicle control does not have such effects. However, one major concern is that PIEZO1 is not only expressed in esophageal tissues, but also expressed in many other cell types including gastric epithelial cells, neuron cells, skin, and red blood cells. It is not clear whether Anti‐PIEZO1‐MMAE could evoke severe side effects when used in vivo. Therefore, we tested the antitumor capacity of Anti‐PIEZO1‐MMAE using a murine xenograft model. Corresponding to the in vitro results, the Ki67 staining in the tumor sections of all groups revealed that Anti‐PIEZO1‐MMAE could efficiently inhibit ESCC cells propagation in vivo. However, one limitation of the current study was due to the original manufacture of ADC reagents stopped their supply, we could not repeat those in vivo experiments with another esophageal cell line and evaluate more biomarkers related to cell proliferation as well as cycle pathways with the tumor tissue samples. Anyhow, we demonstrated that 5‐mg/kg of Anti‐PIEZO1‐MMAE could significantly suppress the tumor growth by inhibiting cell proliferation without any obvious damage to other normal organs and tissues, indicating that targeting PIEZO1 by conjugating its monoclonal antibody with MMAE is a quite feasible and efficient strategy to treat ESCC.

Nevertheless, PIEZO1 was picked when we screened the candidate membrane protein targets with the TE‐1 cell line. It is possible that if we used different cell lines, we might find different targets as the proteome varies greatly among patients and cell lines. Such individual‐specific and cell‐specific proteome screening may provide us with a brand‐new view of personalized therapeutic strategy. In spite of these advantages, some critical limitations of this study should not be overlooked. Firstly, the relatively wide expression of PIEZO1 in multiple human organs could cause unpredictable adverse effect problems. Although we did not observe obvious adverse effects in the treated xenograft mice, whether it is safe for humans requires further investigation. Secondly, the membrane proteome extraction was conducted to include plasma membrane and endomembrane protein components, which may increase the workload of the ADC target screening process. Last but not the least, the significant heterogeneity between patients and cell lines may interfere with the efficiency of ADC target screening and application.

Taken together, this study has uncovered PIEZO1 as a potential ADC target for human ESCC treatment. Targeting PIEZO1 by conjugating its monoclonal antibody with small molecular chemicals such as MMAE can efficiently suppress ESCC tumor cell growth both in vivo and in vitro without inducing any obvious side effects, which represents a promising strategy for improving ESCC patients' prognosis.

## 5 CONCLUSION

This research, by performing membrane proteome screening and validation, has found that the Piezo‐Type Mechanosensitive Ion Channel Component 1 (PIEZO1) is significantly overexpressed in a large proportion (50%) of ESCC patients, and tumor cell surface located PIEZO1 can be internalized with the binding of its monoclonal antibody. Anti‐PIEZO1‐MMAE can induce cell cycle arrest and apoptosis of ESCC cells in vitro and suppress ESCC tumor cell growth in vivo without any obvious side effects. Our study shows that PIEZO1 is a potential antibody‐drug conjugate target for human ESCC.

## CONFLICT OF INTEREST

The authors declare no conflict of interest.

## AUTHOR CONTRIBUTIONS

K.C, W.Z, and S.R. designed and guided the experiments. X.Q. performed the experiments including antibody microarray screening, WB, IHC, IF, qPCR, FACS analysis, cell culture, and xenograft modeling. Z.N. and J.J conducted ESCC patients' data analysis. X.L., X.D., and M.L. helped ESCC patients' sample collection. The manuscript was written by X.Q. and revised by K.C, W.Z, K.M, and S.R.

## ETHICS APPROVAL STATEMENT

All animal and human tissue experiments were approved by the Committee on the Ethics of Animal Experiments of Southern Medical University and Nanfang Hospital, China.

## INFORMED CONSENT STATEMENT

Informed consent was obtained from all subjects involved in the study.

## Supporting information


Figure S1

Figure S2

Figure S3

## Data Availability

Transcriptome sequence datasets of ESCC patients were acquired from the TCGA database at https://www.cancer.gov/about‐nci/organization/ccg/research/structural‐genomics/tcga/studied‐cancers/esophageal. The original data of microarray experiments and ADC screening have not been deposited in public databases. Tissue samples used in the current study were available from the corresponding author upon reasonable request, but the ADC reagents were not available anymore due to the original manufacturer stopped their supply.
