# Supplementary figures and images for: High‐throughput membrane‐anchored proteome screening reveals PIEZO1 as a promising antibody‐drug target for human esophageal squamous cell carcinoma
Source: Cancer Med. 2022 May 24;11(19):3700–13. doi: 10.1002/cam4.4744 (PMC9554447; doi:10.1002/cam4.4744)

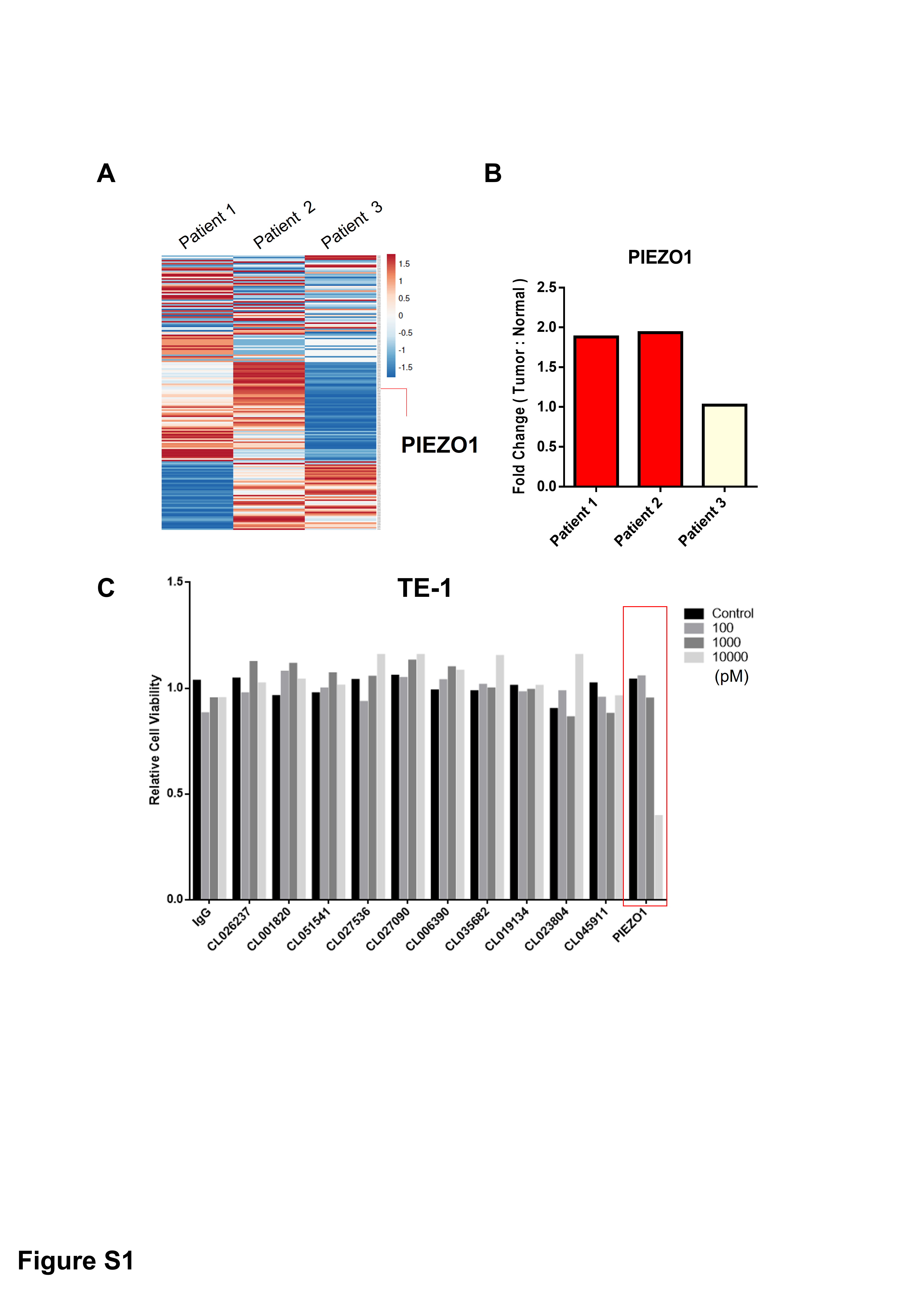

Supplement: Supplementary file 1 — Figure S1 [file CAM4-11-3700-s001.tif]

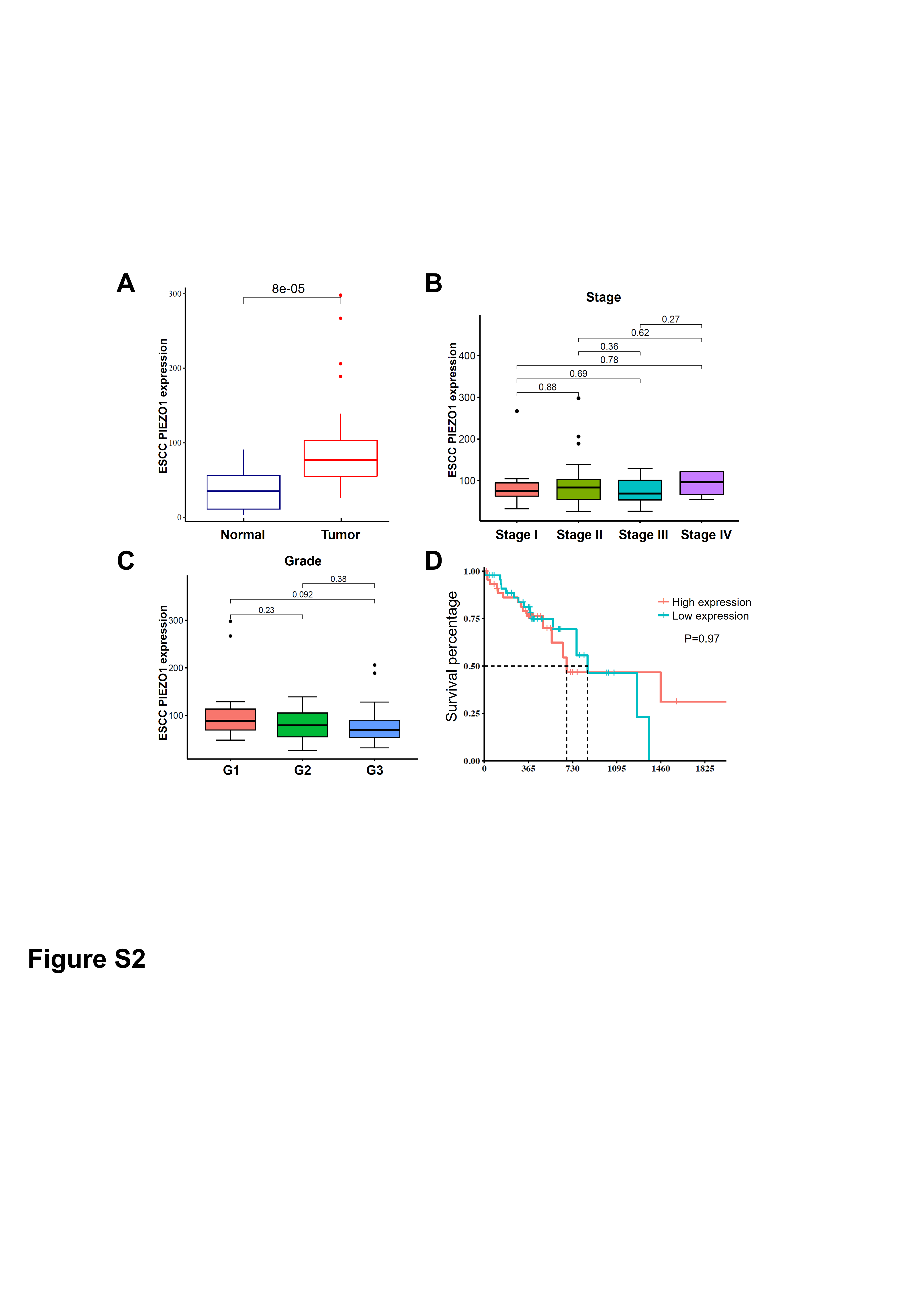

Supplement: Supplementary file 2 — Figure S2 [file CAM4-11-3700-s003.tif]

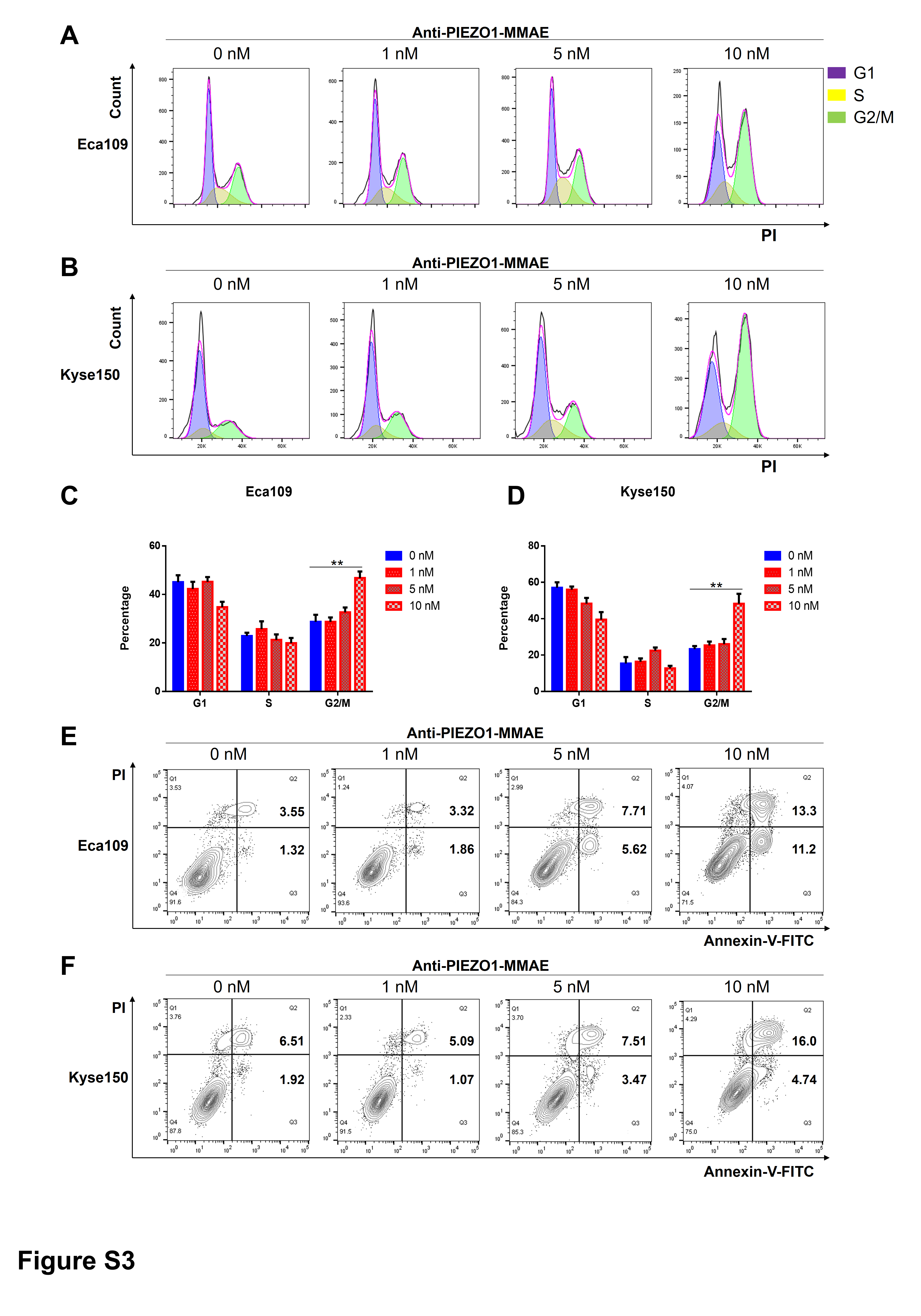

Supplement: Supplementary file 3 — Figure S3 [file CAM4-11-3700-s002.tif]
